# Association between visual problems, insufficient emotional support and urinary incontinence with disability in elderly people living in a poor district in Rio de Janeiro, Brazil: A six-year follow-up study

**DOI:** 10.1371/journal.pone.0217456

**Published:** 2019-05-31

**Authors:** Valéria Teresa Saraiva Lino, Nádia Cristina Pinheiro Rodrigues, Mônica Kramer de Noronha Andrade, Inês Nascimento de Carvalho Reis, Lucília Almeida Elias Lopes, Soraya Atie

**Affiliations:** National Public Health School/ Oswaldo Cruz Foundation, Rio de Janeiro, Brazil; University Medical Center Utrecht, NETHERLANDS

## Abstract

**Introduction:**

Disability follows the rapid rate of population ageing, imposing a huge burden on society. Functional assessment in older people can identify predictors of disability.

**Objective:**

Analyze the incidence and the risk factors for disability in activities of daily living (ADL) and instrumental activities of daily living (IADL) in older adults over six years.

**Methods:**

Six year-follow up study initiated in 2010. The baseline non-probabilistic sample consisted of 180 independent community dwelling individuals aged 60 and over. The procedures comprised an interview with sociodemographic data, questions about falls, urinary incontinence, self-rated health, and assessment of ADL, IADL, mobility, depression, vision, hearing, cognition, nutrition, grip strength and social support. The second research was carried out by telephone and assessed ADL and IADL. Logistic regression models calculated the odds of disability in ADL and IADL according to the age, sex and all other variables.

**Results:**

At six-year follow-up, 118 participants were still alive (65.6%), 31 died (17%) and other 31 were missed (17%). The incidence of disability to performADL and IADL were 25.4% and 32.3%, respectively. The regression logistic models revealed thaturinary incontinence (OR = 3.2; P = 0.03) and insufficient emotional support (OR = 3.8; P = 0.04) were associated with ADL disability, while visual problems (OR: 2.9; P = 0.03) and insufficient emotional support (OR: 5.6; P = 0.01) were associated with IADL disability.

**Conclusion:**

The current study has identified that insufficient emotional support, visual problems and urinary incontinence are associated with disability in older adults. The routine assessment of these problems in the primary care clinics enable the implementation of strategies aimed at reducing or postponing disability. Educating patients and families will also enable better choices to reduce the risk of functional decline.

## Introduction

The demographic transition that has taken place in many countries has produced an aging population that is growing in size and proportion. In Latin America, the accelerated aging process associated with high poverty rates and inefficient health and social security systems imposes to families the most responsibility for the elderly care [1]. The Gini index of income measures the inequality of its distribution, and its value ranges from zero (equality) to one (maximum inequality). In 2015, the Gini index of real average monthly household income per capita in Brazil was 0.485, revealing the inequalities in the income distribution and the concentration of wealth in a small part of the population[2].

As the population gets older, disability becomes a major concern for society. The wide variability in its definition and categorization limits the comparison of frequency measures among countries and studies. For World Health Organization (WHO), disability is an umbrella term for impairment, limitation or participation restriction in activities,resulting from the interaction of health conditions with environmental factors [3]. In a more restricted way, it is related to the need for help from someone else to perform the activities of daily living- ADL, [4] or the instrumental activities of daily living-IADL[5].

Disability rates vary between rich and poor countries. A multicountry study with 49countries evidenced socioeconomic inequality in disability between the rich and poor ones. The median of disability prevalence was higher in the lower income country group (15.1%; 95%CI = 13.7, 18.8%) than in the higher income country group (10.8%; 95% CI = 6.5%, 13.4%). But in all study countries, disability was more prevalent in the poorest than in the richest wealth quintile[6].

Although increasing age is by far the most important predictor of disability, other factors contribute to this condition. Socioeconomic aspects related to poverty determine more unfavorable health outcomes, lower educational achievement and increased rates of disability[3]. In addition, the presence of comorbidities and neuropsychiatric conditions, lack of physical activity and extreme values of body mass index are significant predictors of functional decline[7, 8]. Social support (SS) influences physical health outcomes among older adultsandis also associated with disabilityand mortality. The perceived support, more than the received one, has been linked to beneficial health outcomes [9].

To face disability, individuals and society will benefit with a shift from reactive care to proactive care [10]. Such a change of procedure will enable the implementation of prevention strategies, which includes interventions on clinical, psychological and social determinants of the elderly health. The objective of this study was to analyze the disability incidence in ADL/ IADL and identify the predictive factors for functional decline over six years in a community-dwelling older adults sample residing in a poor region in Rio de Janeiro, Brazil.

## Materials and methods

### Study setting and sample

This is a six-year follow up study. The baseline and follow-up studies are described below.

### Baseline study

Between June and December 2010 we enrolled 180 elderly men and women in a cross-sectional study aimed at elaborating a functional screening strategy based on a comprehensive geriatric assessment[11]. The research was performed at the primary care unit of the Oswaldo Cruz Foundation, in the city of Rio de Janeiro, Brazil. At that time, the unit provided services to 31,000 residents of the Manguinhos District, where most houses had a single room, sanitary issues were still unsolved, monthly family income was usually lower than the Brazilian minimum wage (US$ 295) and more than 50% of residents had no more than elementary school education[12]. Initiatives aimed at decreasing violence had been impeded by the activities of local drug traffickers[13].

Participants were eligible if they were aged 60 and over, physically and mentally able to visit the health center independently. The non-probabilistic sample was drawn from users who received care from the Family Health Team. We calculated the sample size using as reference the prevalence of depression, a common disorder in older adults, estimated in 20%[14]. A kappa coefficient of 0.6 with a 95% confidence interval was used to generate a conservative sample size of 180 individuals, estimated using the Win- Pepi application, version 2009[15].

### Procedures at baseline study

The procedures comprised an interview with sociodemographic data (education was measured in terms of years of schooling), questions about falls, urinary incontinence (UI), self-rated health (SRH) and the assessment of ten functions. Activities of daily living (ADL) and the Instrumental Activities of Daily Living (IADL) were investigated in the baseline and in the follow-up study with the same tools. The Katz ADL scale assesses six activities: bathing, dressing, toileting, transferring, continence and feeding. Disability implies in needing help to perform any activity. Each question is scored as zero (not impaired) or one (impaired), generating a score, where zero and six represent complete independence and dependence, respectively[4, 16]. The *Functional Activities Questionnaire* (Pfeffer scale) assesses the degree of independence to perform IADL on a scale of 11items. The score ranges from zero (able) to three (unable) in each item, so that the higher the final score, the greater is the patient’s dependence[5]. The other functions were: mobility (*Timed Up and Go*)[17], depression (*Structured Clinical Interview for DSM-IV)*[18], vision (Snellen card)[19], hearing (whispered test)[20], cognition (*Mini-Mental State Exam*)[21, 22], nutrition (Body Mass Index-BMI)[23], grip strength[24] and social support (*Medical Outcomes Study Social Support Scale*-MOS). Three out of five dimensions (material and emotional support and social interaction) of MOS scale’s Brazilian version were selected due to their importance to the elderly. Social support was classified as sufficient or insufficient[25–27]. Chronic diseases were checked in medical records. Information about cut-off points can be found in the supplementary material.

### Follow-up study

The follow-up survey was conducted by telephone with individuals who were still alive and mentally and physically able to respond, or their family proxies. Data were collected from September to December 2016. The dependent variables were ADL or IADL disability. The risk factors were selected based on a literature review and the availability of data in our sample at baseline.

### Statistical analysis

Summary measures of numerical variables, frequency of categorical variables and incidence of disability in ADL and IADL in six years were calculated. Through two logistic regression models, we investigated the odds of disability for ADL and IADL (dependent variables) according to age, sex and functionality variables. In order to specify the models, we included all the variables that presented a p-value lower than 0.20 in bivariate analysis. In the first, dependent variable was incidence of IADL from 2010–2016 and the independent variables were sex, age, BMI, study years, mobility, grip strength, depression, visual problems, UI, and insufficient emotional support. In the second model, dependent variable was incidence of ADL from 2010–2016 and the independent variables were sex, study years, grip strength, UI, insufficient emotional support and Parkinson disease.

The generalized variance-inflation factor and Hosmer and Lemeshow goodness of fit (GOF) test were calculated for both models. K-fold cross-validation methods were used to evaluate the models performance on different subset of the training data (k = 10)[28].

The software R-Project version 3.5.1 was used for the data analyze.

### Ethics

The Ethics Research Committee of the National Public Health School/Fiocruz) approved both the researches (report numbers 126/2010; 392/ 2015). All participants signed an informed consent form, which pledges anonymity and confidentiality of the information before the baseline and follow-up studies.

## Results

In the group of 180 participants examined in 2010, the average age was 73.1 (SD 7.0) and the overall majority were women (73.3%). At six-year follow-up, 118 participants accepted to participate (65.6%), 31 were dead (17%) and other 31 had been missed (17%). Reasons for non-participation in the follow-up were the change of address (16.1%) and lack of interest (1.1%). There were no statistically significant differences between the characteristics of the studied population and the missing group, except for a higher proportion of individuals who reported insufficient social interaction in the missinggroup.

The incidence of disability in ADL and IADL over six years were 25.4% and 32.2%, respectively. For both outcomes, UI and insufficient emotional support presented statistical and borderline significance, respectively. Study years and BMI reached statistical significance only for IADL, while female grip strength presented statistical significance for ADL (Table 1).

**Table 1:** Baseline characteristics of the study population and the bivariableassociations between potential predictors of disability in activities of daily living and instrumental activities of daily living, 2010–2016.

Factors	Groups	Total	IADL	P-value	ADL	P-value
**Sex–n (%)**	Female	132 (73.3)	32 (36.0)	0.17	26 (29.2)	0.14
	Male	48 (26.7)	6 (20.7)		4 (13.8)	
**Age–n (%)**	≤ 73	88 (48.9)	15 (25.0)	0.12	16 (26.7)	0.88
	>73	92 (51.1)	23 (39.7)		14 (24.10)	
**Marital–n (%)**	Married	81 (45.0)	14 (26.9)	0.32	12 (23.1)	0.67
	Unmarried	99 (55.0)	24 (36.4)		18 (27.3)	
**Study years ≥ 5**	Yes	49 (27.2)	4 (12.5)	0.007	5 (15.6)	0.16
	No	131 (72.8)	34 (39.5)		25 (29.1)	
**Mobility–n (%)**	Without mobility disorder	68 (38.2)	10 (21.7)	0.11	12 (26.1)	0.99
	Acceptable	99 (55.6)	26 (39.4)		17 (25.8)	
	Mobility disorder	11 (6.2)	2 (40.0)		1 (20.0)	
**BMI (%)**	Overweight	105 (59.0)	26 (35.1)	0.05	22 (29.7)	0.48
	Eutrophic	59 (33.1)	6 (18.8)		6 (18.8)	
	Low weight	14 (7.9)	6 (60.0)		2 (20.0)	
**Grip strength–n (%)**	Inadequate	56 (31.2)	14 (40.0)	0.28	6 (17.1)	0.25
	Adequate	123 (68.7)	24 (28.9)		24 (28.9)	
Male grip strength–n(%)	Inadequate	19 (39.6)	4 (40.0)	0.14	3 (30.0)	0.10•
	Adequate	29 (60.4)	2 (10.5)		1 (5.3)	
Female grip strength–n(%)	Inadequate	37 (28.2)	10 (40.0)	0.63	3 (12.0)	0.04*
	Adequate	94 (71.8)	22 (34.4)		23 (35.9)	
**Falls–n (%)**	Yes	42 (23.3)	12 (40.0)	0.37	10 (33.3)	0.33
	No	138 (76.7)	26 (29.5)		20 (22.7)	
**Visual problems–n (%)**	Yes	82 (46.3)	24 (45.3)	0.13	14 (26.4)	0.99
	No	95 (53.7)	14 (22.2)		16 (25.4)	
**Auditory deficit–n (%)**	Yes	27 (15.0)	6 (35.3)	0.78	5 (29.4)	0.76
	No	153 (85.0)	32 (31.7)		25 (24.8)	
**UI**	Yes	40 (22.2)	12 (54.5)	0.02	10 (45.5)	0.03*
	No	140 (77.8)	26 (27.1)		20 (20.8)	
**Self-perceived health**	Regular/Bad/Very bad	20 (11.1)	4 (36.4)	0.74	3 (27.3)	0.99
	Good/Very good	160 (88.9)	34 (31.8)		27 (25.2)	
**MMSE**	Inadequate	66 (36.7)	14 (38.9)	0.39	8 (22.2)	0.65
	Normal	114 (63.3)	24 (29.3)		22 (26.8)	
**Emotional Support**	Insufficient	22 (12.3)	8 (53.3)	0.08	7 (46.7)	0.06•
	Adequate	157 (87.7)	30 (29.4)		23 (22.5)	
**Material Support**	Insufficient	13 (7.3)	4 (33.3)	0.99	4 (33.3)	0.50
	Adequate	166 (92.7)	34 (32.4)		26 (24.8)	
**Social Interaction**	Insufficient	36 (20.1)	5 (23.8)	0.44	6 (28.6)	0.78
	Adequate	143 (79.9)	33 (34.4)		24 (25.0)	
**Depression**	Yes	52 (28.9)	13 (41.9)	0.19	10 (32.3)	0.34
	No	128 (71.1)	25 (28.7)		20 (23.0)	
**Number of comorbidities**	≥3	103 (57.2)	19 (30.2)	0.69	19 (30.2)	0.29
	<3	77 (42.8)	19 (34.5)		11 (20.0)	
**Cataract**	Yes	88 (48.9)	19 (35.8)	0.55	15 (28.8)	0.53
	No	92 (51.1)	19 (29.2)		15 (23.1)	
**Parkinson**	Yes	4 (2.2)	2 (66.7)	0.24	2 (66.7)	0.16
	No	176 (97.8)	36 (31.3)		28 (24.3)	
**Diabetes**	Yes	57 (31.7)	8 (25.0)	0.38	7 (21.9)	0.64
	No	123 (58.3)	30 (34.9)		23 (26.7)	
**Hypertension**	Yes	150 (83.3)	31 (32.0)	0.99	27 (27.8)	0.27
	No	30 (16.7)	7 (33.3)		3 (14.3)	
**CAD**	Yes	18 (1.0)	5 (38.5)	0.75	4 (30.8)	0.74
	No	162 (9.0)	33 (31.4)		26 (24.8)	
**COPD**	Yes	21 (11.7)	3 (17.6)	0.26	5 (29.4)	0.76
	No	159 (88.3)	35 (34.7)		25 (24.8)	
**Asthma**	Yes	13 (7.2)	3 (33.3)	0.99	3 (33.3)	0.69
	No	167 (92.8)	35 (32.1)		27 (24.8)	
**Osteoarthritis**	Yes	77 (42.8)	18 (32.7)	0.99	15 (27.3)	0.68
	No	103 (57.2)	20 (31.7)		15 (23.8)	

BMI- Body Mass Index; CAD- coronary artery disease; COPD- chronic obstructive pulmonary disease; MMSE- Mini-Mental State Exam; UI- urinary incontinence.

* Statistical significance (p-value < 0.05);

^•^ borderline statistical significance (p-value<0.10).

The regression logistic models revealed that UI and insufficient emotional support were associated with ADL disability while visual problems and insufficient emotional support were associated with IADL disability (Table 2). The generalized variance-inflation factors ranged from 1.10 to 1.36 for the adjusted model that explain the IADL occurrence, and from 1.02 to 1.09 for the adjusted model that explain the ADL occurrence. P-values of Hosmer and Lemeshow goodness of fit (GOF) tests were 0.47 and 0.97 for the adjusted models that explain the IADL and ADL occurrence, respectively. The RMSE (K-fold cross-validation) for the adjusted models that explain the IADL and ADL occurrence were 0.45 and 0.42 respectively.

**Table 2:** Associations between potential predictors of disability in activities of daily living and instrumental activities of daily living,2010–2016.

Factors	IADL OR	P-value	ADL OR	P-value
**Sex**	2.2	0.19	2.6	0.14
**Age**	1.1	0.18		
**BMI**	1.1	0.10		
**Study years ≥ 5**	0.4	0.19	0.5	0.23
**Mobility**	1.1	0.18		
**Strength**	0.9	0.83	2.1	0.17
**Depression**	1.2	0.78		
**Visual problems**	2.9	0.03		
**UI**	2.1	0.22	3.2	0.03
**Insufficient Emotional Support**	5.6	0.01	3.8	0.04
**Parkinson**			11.0	0.08

ADL- Activities of Daily Living; BMI = Body Mass Index; IADL- Instrumental Activities of Daily Living; OR = Odds Ratio; UI- urinary incontinence.

## Discussion

This study identified a high incidence of ADL/IADL disability in poor elderly over six years, mainly due to insufficient emotional support, UI and visual problems. The incidence of ADL/IADL disability was 25% and 32%, respectively. These high rates of disability align with previous findings. Among older Brazilians, reduced disability is associated with higher income as evidenced in a study that examined income-disability relationships with more than 63,000 older adults. There was a strong and predominantly linear relationship between higher income and lower prevalence of disability. For those in the 50^th^ percentile or under this level, the prevalence of disability was around 21% and 33% for men and women, respectively. For those between the 86-90^th^ percentiles the prevalence rates decreased to 11% and 26% for men and women, respectively[29].

The most surprising information that came up with this research was the huge increase in disability associated to insufficient emotional support. It raised almost four and six times the risk of ADL and IADL disability over six years, respectively. This finding is in line with other studies. A three-year follow–up study also evidenced higher risk of disability in Danish elderly who were not satisfied with their social relationships when compared with those who were very satisfied (OR = 1.70; 95% CI 1.30–2.24)[30]. McLaughlin *et al* demonstrated that social network size was not associated with disability, but rather the subjective perception of SS in a follow up Australian study with elderly men and women (OR = 1.5; 95% CI 1.27–1.84 for women; OR = 1.52 95% CI 1.11–2.08 for men)[31]. Lonely subjects were also more likely to experience decline in ADL over six years in United States (24.8% vs 12.5%; RR = 1.59; 95% CI, 1.23–2.07)[32] while in India, the feeling of loneliness increased the odds of ADL disability more than twice (OR = 2.3; 95% CI: 1.0–5.3)[33]. In Brazil, a six-year follow-up study focused more on chronic diseases, socioeconomic, demographic and lifestyle aspects than on SS as risk factors for functional decline[34] but data from cross-sectional studies reveal higher chances of disability in older adults who don’t visit friendsand relatives and in those who report poor SS[35–37].

The influence of SS on physical health outcomes is well documented. Low SS is a risk factor for mortality comparable to other well-established ones and is related to morbidity and mortality, especially for cardiovascular diseases [9, 38]. Social frailty, identified through questions about social activities, roles and relationships, is associatedwith disability incidence[39].

The causal pathways through which SS protects against disability are likely multifactorial but the link between SS and several related inflammatory diseases can be partially explained in the same way that mammalian respond to acute stress. In these animals, the brain process threats to social connection in a manner similar to acute threats to survival, thus activating the sympathetic nervous system (implicated in the fight-or-flight response) and the hypothalamic-pituitary-adrenal axis. Low social connection induces prolonged stress response, raising inflammatory activity and contributing to the development of various inflammatory-related diseases as well as to increased mortality[40].

Uchino has proposed that different aspects of SS (received and perceived) are related to health in a life-span perspective. Perceived support relatesmore to beneficial health outcomes than received support. Individuals with positive early family environments (parental support, less conflict) develop psychosocial profiles that enable better coping with life stressors, healthier behavior choices and lower stress exposure. All these factors may favor lower rates of chronic disease development and less disability in the old age [9]. It is possible that people who have been living in environments with high levels of poverty and violence, like those in this study, may not have developed favorable skills needed to perceive SS as satisfactory and relevant, enabling the creation of emotional ties between individuals. Interventions aimed at improving SS should take into account all the aspects mentioned before. They will require additional approaches (eg, cognitive-behavioral therapies) that guide the perception of received support in a satisfactory and healthy direction [9]. Considering only the size of the social network can be insufficientto limit subsequent disability [31].

Another risk factor for disability evidenced in this study was UI, a common geriatric syndrome that usually affects self-esteem and quality of life more than functional capacity[41]. Those who reported UI had risk for disability more than three times higher than the elderly who did not report UI. The association between UI and disability can be found in previous studies. American elderly women with UI had 3.31 increased odds of functional difficulty or dependence compared with those without UI, even after adjusting for sociodemographic factors and parity[42]. ADL disability was also present in 26% and 30% of incontinent men and women, respectively, in a survey carried out with more than 43,000 elderly rural villagers in Bangladesh. In these villages, the toilet facilities are outside the main living quarters, a fact that can impair the access of those with poor mobility and increase the prevalence of UI[43]. These findings suggest the need to prevent disability in elderly people with UI.

The third risk factor for disability identified in this study was visual problem, a highly prevalent condition in old age. The risk of IADL disability for the visually impaired elderly was almost three times higher than for those non-visually impaired over six years. The association of low vision with disability is well documented[7]. Other studies corroborate our findings. Rosso*et al* evidenced risk 1.49 times greater (95% IC 1.17–1.89) of disability in older American women with low vision in a three-year follow-up study with almost 30 thousand persons [44]. Visual impairment was the most common cause of disability reported in Brazilian population in 2010 with a prevalence estimated in 18.8%[45]. In Brazil, a cross-sectional study with 580 individuals reported higher prevalence of disability and poor quality of life in a group of elderly with subnormal vision or blindness when compared with those with normal vision (p<0.001)[46]. The early identification and treatment of vision disorders can be a measure of high impact in maintaining the independence of the elderly, especially in poor regions.

This study presents limitations and strengths. Sociodemographic and socioeconomic characteristics are associated to disability. Higher age, female gender, having comorbidity, living in an urban area, fewer years of education and lower income level have been reportedas risk factors for developing limitations in ADL[47, 48]. All these aspects were present in our sample and probably contributed to the high incidence of disability revealed here. The lack of a statistically significant association between disability and these factors is probably due to the limited number of the sample at the end of the six-year study period. Other Brazilian studies have demonstrated increased incidence and prevalence of disability associated to female gender, age, schooling, income leveland living conditions[34, 49, 50]. Other limitation is that only one follow-up data collection was performed. Further data collection over these 6 years could increase the statistical power of the study, making it possible to identify associations of smaller magnitude. Nevertheless, it was possible to demonstrate that poor SS, UI and visual problems were predictors of disability in this population. As the design of the baseline study was aimed at elaborating a strategy for geriatric assessment at primary care settings, lifestyle-related information were not investigated. Physical exercise can slow down functional decline [51]. We recognize that its effect on disability unaccounted could potentially introduce a bias in the study, but we assessed handgrip strength, a measure of physical performance, and it was not associated with disability. Thus, we can suppose that the introduction of a bias would underestimate the strength of association between visual problems, insufficient emotional support and urinary incontinence with disability. Other limitation is the use of a convenience sample of older adults from a primary care setting. Although the results can’t be generalized, these individuals are similar to those living in poor metropolitan areas in Brazil. In addition, we used tools that were validated in Brazil and we selected a sample of unimpaired elders, making adjustments for confounding factors according to a series of variables, resulting in true associations. Although the present study used imbalanced data to verify the association between health problems and disability in elderly people, future studies concerned in building prediction models, especially using machine learning models, could be used, as recent work has demonstrated in the context of hospital associated disability [52]. Finally, this is one of the longest follow up study studies about risk factors for disability conducted in Brazil. The six-year follow-up makes it possible to draw conclusions about the causes of disability.

## Conclusion

The rapid rate of population ageing will bring a tremendous social and financial impact upon society and the health system. Disability follows the rhythm of the ageing process, making the oldest ones more prone to functional decline. This imposes a huge burden on families and caregivers. The challenge for the health system is to propose strategies to postpone or prevent functional decline. The current study has identified risk factors associated with increased disability incidence in the elderly, which may be modifiable. Insufficient emotional support, visual problems and UI are easily measured predictors for disability. The periodic screening of these problems during routine visits to the primary care clinics will probably enable the implementation of strategies and treatments aimed at reducing or postponing disability. Educating patients and families will also allow better choices to reduce the risk of functional decline. Studies with poor populations are necessary to verify the effects of interventions that reduce the impacts of insufficient emotional support, visual problems, and UI on the high incidence of disability in the elderly.

## Supporting information

S1 File Procedures at baseline study. (DOC)
