# Supplementary material for: Association between visual problems, insufficient emotional support and urinary incontinence with disability in elderly people living in a poor district in Rio de Janeiro, Brazil: A six-year follow-up study
Source: PLoS One. 2019 May 31;14(5):e0217456. doi: 10.1371/journal.pone.0217456 (PMC6544343; doi:10.1371/journal.pone.0217456)
Supplement: S1 File — (DOC) [file pone.0217456.s001.doc]

Procedures at baseline study

| Function | Tool | Cut-off |
| --- | --- | --- |
| Mobility | Timed Up and Go ^1^ | Mobility disturbance if > 20 seconds. |
| Depression | Structured Clinical Interview (SCI) for DSM-IV Axis I Disorders – Clinician Version^2,3^ | SCI follows an interview script. At least five questions, in 10, with affirmative answers for depression. One of them must be referring to loss of interest or sadness. |
| Vision | Snellen card^4^ | Visual impairment< 20/40 |
| Hearing | Whisperedtest^5^ | Hearingimpairment- failure in answering a question in one ear. |
| Cognition | Mini-Mental State Exam^6,7^ | Illiterates-19; less than 8 years of schooling- 23; ≥8 years of schooling-26. |
| Nutrition | Body Mass Index^8^ | Risk of nutritional disturbance- BMI < 21Kg/m^2^ body surface. |
| Gripstrength | Dynamometer – levels of grip strength measured according to Fried’s criteria for frailty.9 | |  | BMI | Normal Strength (Kg) | | --- | --- | --- | | Men | ≤ 24 | > 29 | | ≤24,1 - 28 | > 30 | | ≥ 28 | > 32 | |  |  |  | | Women | ≤ 23 | > 17 | |  | 23,1 - 26 | > 17,3 | |  | 26,1 - 29 | <18 | |  | > 29 | ≤ 21 | |
| Socialsupport | Medical Outcomes Study Social Support Scale-MOSsocial interaction, material and emotional support.^10,11,12^ | The scale contained four questions for each type of support totaling 12 questions, with answers ranging from never (1 point) to always (5 points). The sum of all answers determined the SS score, which ranged from 12 to 60 points. Satisfactory SS ≥ 48;unsatisfactory SS≤47. |

1. Podsiadlo D, Richardson S. The timed "Up & Go": a test of basic functional mobility for frail elderly persons. J Am Geriatr Soc. 1991;39(2):142-8.
2. First MB, Spitzer RL, Gibbon M, Williams JBW. Structured clinical interview for DSM-IV axis I disorders- clinician version (SCID-CV) Washington (DC): American Psychiatry Press; 1997.p. 84.
3. Del- Ben CM, Vilela JAA, Crippa JAS, Hallak JEC, Labate CM, Zuardi AW. Rev. Bras. Psiquiatr 2001, 23; (3): 156-159. Confiabilidade da "Entrevista Clínica Estruturada para o DSM-IV – Versão Clínica" traduzida para o português
4. Mangione C. Vision. In: Osterweil D, Brummel-Smith K, Beck J, editors. Comprehensive Geriatric Assessment. New York: Mc Graw Hill; 2000. p. 285-94.
5. Pirozzo S, Papinczak T, Glasziou P. Whispered voice test for screening for hearing impairment in adults and children: systematic review. BMJ. 2003;327(7421):967.
6. Bertolucci PH, Brucki SM, Campacci SR, Juliano Y. The Mini-Mental State Examination in a general population: impact of educational status. Arq Neuropsiquiatr. 1994;52(1):1-7.
7. Almeida OP. Mini mental state examination and the diagnosis of dementia in Brazil. Arq Neuropsiquiatr. 1998;56(3B):605-12.
8. Lipschitz DA. Screening for nutritional status in the elderly. Prim Care. 1994;21(1):55-67.
9. Fried LP, Tangen CM, Walston J, Newman AB, Hirsch C, Gottdiener J, Seeman T, Tracy R, Kop WJ, Burke G, McBurnie MA. Frailty in Older Adults: Evidence for a Phenotype. J.Gerontol 2001; 56 (3): M146-56.
10. Sherbourne CD, Stewart AL. The MOS social support survey. Soc Sci Med. 1991;32(6):705-14.
11. Griep R. Confiabilidade e Validade de Instrumentos de Medida de Rede Social e de Apoio Social Utilizados no Estudo Pró-Saúde. Rio de Janeiro: Escola Nacional de Saúde Pública Sergio Arouca.; 2003
12. Lino VT, Portela MC, Camacho LA, Atie S, Lima MJ.Assessment of social support and its association to depression, self-perceived health and chronic diseases in elderly individuals residing in an area of poverty and social vulnerability in Rio de Janeiro city, Brazil. PLoS One. 2013 Aug 12;8(8):e71712. doi: 10.1371/journal.pone.0071712. eCollection 2013.
